# Developing a secure base in family intervention: using the adult attachment projective system to assess attachment in family relationships

**DOI:** 10.3389/fpsyg.2023.1291661

**Published:** 2023-11-03

**Authors:** Carol George, Julie Wargo Aikins

**Affiliations:** ^1^Mills College at Northeastern University, Oakland, CA, United States; ^2^Merill Palmer Skillman Institute and The Department of Psychiatry and Behavioral Neuroscience, Wayne State University, Detroit, MI, United States

**Keywords:** attachment, representation, adult attachment projective picture system, parents, trauma, family therapy

## Abstract

Families are core to human well-being. Therapeutic intervention may be needed in the context of family disruptions. Attachment theory conceptualizes parents as the secure base and safe haven that support children’s optimal development. Parents who have experienced their own attachment difficulties or traumas may not provide quality caregiving necessary for balanced secure parent–child attachment relationships. Following Bowlby’s original thinking (1988), an attachment approach to family intervention views the therapist as a secure base that enables families to explore individual and system problems to restore equilibrium. Attachment informed therapy uses attachment theory to understand family functioning. However, the unavailability of valid economical assessment for examining attachment representations has constricted the practical utility of attachment theory in family therapy beyond applications of general concepts. This chapter describes the Adult Attachment Projective Pictures System (AAP) and explores its use as an efficient manner for assessing attachment representations within families that allows therapists to understand problematic interactions, disabling defensive processes, make predictions concerning negative patterns, and create targets for change and restorative intervention. Consolidating three decades of attachment and caregiving system research, we describe how distinct patterns of AAP responses for each adult attachment group map onto expected parenting and family system expectations and behaviors to provide a concise and informative framework. In addition to the traditional adult attachment patterns (Secure, Dismissing, Preoccupied, Unresolved), we describe for the first time expectations for two additional forms of incomplete pathological mourning (Failed Mourning and Preoccupied with Personal Suffering) that have been overlooked in the field.

## Introduction

Families are the center of human life. Establishing, sustaining, and mourning relationships past and present are essential family themes. The attachment aim of family intervention is to create a secure base. In attachment theory, the secure base is the foundation of security and survival that encourages children to reach out for protection when they are distressed (Bowlby, 1988; Ainsworth and Bowlby, 1991). When applied to families, the secure base is the network of resources from which the family can explore new solutions to family problems with shared awareness of availability and collaboration (e.g., Byng-Hall, 1995; Kim et al., 2018). Optimally, parents support children’s security when they are committed, sensitive, responsive, flexible, consistent, and thoughtful about their and their children’s internal states (George and Solomon, 2008, 2016). Parent–child attachment relationships regulate neurophysiological systems of emotion-, stress-, and self-regulation (Schore and Schore, 2008). The emphasis on caregiving behavior, parents’ state of mind (also termed “representation”), and the conceptual emphasis on affect and affect regulation provides a pragmatic framework for change “rooted in dynamic relational processes” (Schore and Schore, 2008; Diamond et al., 2021) and helps refocus therapy from a single problem source to the family.

Today, many family therapists are familiar with and may implement an attachment lens in their practice (Byng-Hall, 2006; Diamond et al., 2021). Attachment-based goals are consistent with long-standing approaches to family therapy (Johnson and Whiffen, 2003). Attachment theory provides the groundwork for conceptualizing family difficulties and deficits rather than introducing new therapeutic approaches for family therapy (e.g., family system therapy). At the individual and family levels, attachment’s unique contribution to the therapeutic milieu is its conceptual roots in evolutionary biology. It provides an organic context for families to explore tension, conflict, and fear in ways that regulate emotional distress and reduce shame that can threaten family emotional-communication bonds; this exploration is fundamental to basic human survival. Clients often feel relief when they learn that their discomfort, anger, and shame are biologically based and not irrational products of their imaginations.

Although familiar, most therapists have yet to be trained to apply the depth and complexity of attachment ideas to family intervention. Even fewer know how to access reliable, valid assessments that uncover the depth of unconscious processes rooted in each family member’s representational attachment state of mind. Each family member brings a different script or representational map about how relationships work and stressful triggers. Parents tend to replicate their attachment patterns because this is what they know. They typically do not constructively think about and revise their representation of the past to meet the present challenges, especially when their past is laden with trauma. Knowing about individual attachment patterns can help therapists understand and create an empathic environment for family members and help solve family problems by predicting and pre-empting responses to family situations or therapy (Byng-Hall, 1995).

Unique to applying attachment theory to therapeutic intervention is the notion that the therapist is a secure base for the family. Through this role, not only can the therapist serve as a trusting, sensitive, and responsive figure that may differ from family members’ previous experiences, but also the therapist may model for parents how they may fill these roles for each other and their children. By serving as a secure base, therapists promote exploring difficulties within therapy. Without the safety of these therapist-family relationships, family members may not be able to examine the obstacles within their families or how their own attachment representations are re-enacted and transmitted in their current family. Over time, once new ways of relating and problem-solving have been established, the safety and security provided by the therapist can be transferred to the network of family relationships.

The chapter begins by conceptualizing the family from an attachment theory perspective, addressing the function and goal of attachment relationships. Here, we also define attachment trauma, which is poorly explained in most attachment literature, and discuss how past trauma can subvert the family as a secure base. We next introduce the *Adult Attachment Projective Picture System* (AAP, George and West, 2012; George et al., 2023) as a rich, valid, and economical assessment for family intervention. We next present the nuances of unique representational maps, describing individuals’ defenses, expectations, and anticipated interaction patterns. The discussion focuses on the parents in the family system. One may also use the AAP with adolescents to uncover the map they contribute to family processes.

## Conceptualizing the family in terms of attachment

Understanding one another’s feelings, motivations, and behaviors within family life contributes significantly to family interactions (Byng-Hall, 2006). As such, conceptualizing the family using an attachment lens is not a new idea. Attachment is a systems theory and families reflect a system of interactions (Stevenson-Hinde, 1990). The family develops a shared working model encompassing individual and family rules for how members think and behave.

At the core, attachment begins with the child-caregiver relationship and, by adolescence, is a fundamental facet of one’s identity as a person who feels worthy of protection and care and able to protect others (George and Solomon, 2008; Aikins et al., 2009). Attachment is an inherited “organized behavioral system” that interacts with other fundamental inherited systems, such as fear and exploration or peer relationships, to ensure one gets the protection needed to survive and create a new family (Bowlby, 1982). The ultimate function of attachment, then, is survival; the goal in everyday life is to stay close to or be able to summon the persons who are responsible for your protection (Bowlby, 1982). The *attachment system* is complemented by the parents’ *caregiving system* that, in tandem, achieves these goals (Bowlby, 1982; George and Solomon, 2008). The function of the caregiving system is to protect children with the goal of keeping children close enough to keep them safe (George and Solomon, 2008).

The attachment system does not exist in isolation. Just as the attachment system is juxtaposed with the caregiving system, these systems are placed within the broader context of the family (Stevenson-Hinde, 1990). Each dyad within the family is a subsystem of this larger framework. Dyads develop patterns of adaptive or maladaptive behavior and communication that become representational maps of the self in relation to others and the rules for sustaining these relationships (Bowlby, 1982). As such, Stevenson-Hinde (1990) stressed that therapeutic targets in family systems therapy models include “allocation of roles, behavior control, problem-solving, communication, affective responsiveness, affective involvement, and overall functioning {are related} to patterns of attachment” (p. 224) in part reflecting that patterns developed during childhood tend to be recycled and perpetuate (Bretherton and Munholland, 2016).

One feature that is especially subject to perpetuation is attachment trauma. Called *intergenerational transmission,* parents can unconsciously perpetuate the interactions and effects of their own traumatic experiences even when they are working very hard not to do so. This is because trauma is not just an experience. Indeed, actual behavior may not be replicated. What is transmitted from parent to child is an attachment trauma state of mind. The dictionary defines trauma as a violently produced physical or psychological wound accompanied by shock (Webster’s dictionary). In psychiatry, trauma is partly determined by the enduring emotional effects of shock and alarm, including chronic debilitating anxiety, fear, and anger. The attachment theory approach is narrower, beginning historically with Bowlby’s discussion of loss (Bowlby, 1980; Main and Solomon, 1986; Main and Hesse, 1990).

The past three decades of attachment research have focused almost unilaterally on trauma, defined as loss or abuse. Of particular interest to clinicians, however, are individuals who show signs of trauma but not experienced loss or abuse. Instead, clients describe experiences clinicians often call “little t trauma” (e.g., divorce, emotional abuse, chronic parental mis-attunement, parental chemical dependency). What all forms of attachment trauma have in common is threats to the self or the attachment-caregiving relationship that risks leaving the child vulnerable and unprotected (Solomon and George, 2011; George and West, 2012). Attachment trauma “assaults” the child’s biological need for protection. Children who experience enduring “failures” in parental protection become dysregulated, chronically frightened, and determined to find ways to protect themselves or feel they will perish (George and Solomon, 2008). Consequently, failed protection by attachment figures, regardless of whether there is experience of abuse or loss, compromises psychological safety, self-integrity, and ultimately, survival. These children feel physically or psychologically abandoned in those frightening moments when they need protection the most (Solomon and George, 2011; George and Solomon, 2016).

Family intervention using attachment concepts benefits from moving beyond general concepts and therapeutic guesswork about attachment patterns to developing a thorough understanding of the nuances of different attachment patterns, especially for patterns where trauma is involved. Ideas about the role of attachment in family intervention date back over three decades. Why, then, has attachment remained on the periphery for many family clinicians? Why have not attachment-minded therapists been able to integrate the rich variations in the attachment maps of clients with different attachment patterns in their work? We have found that a significant problem clinicians encounter in family intervention settings is assessment. Readers familiar with the family systems literature are likely acquainted with discussions of the *Adult Attachment Interview* (AAI, George et al., 1985; Main and Goldwyn, 1998). The AAI is an interview that designates a client’s attachment pattern from the interview narrative about experiences and thoughts about the past. Unfortunately, it is impractical for most clinical use. It is expensive, cumbersome to learn and implement, and does not assess a client’s full scope of attachment trauma experiences (interview questions are limited to loss and abuse). The assessment-minded reader may also have been interested in paper and pencil attachment self-report tests. Self-report measures assess social cognitions. These are subject to positive self-report bias where reported attachment dimensions, which for many clients, are sabotaged by unconscious defensive processes (e.g., Riggs et al., 2007). Further, extensive empirical scrutiny of self-reports shows that the assessment findings are poorly related to childhood experience (George and West, 2012; George et al., 2023). This body of work shows then that the AAI and self-report measures are easily subject to withholding trauma (Spieker et al., 2011).

The following section summarizes the AAP, a clinically friendly, economical alternative to the AAI that delves deeper into attachment trauma than any other available attachment assessments. The reader who would like more information about the AAP than described here is referred to two comprehensive volumes that discuss the development, validation, and scoring and classification system (George and West, 2012; George et al., 2023).

## The Adult Attachment Projective Picture System (AAP)

Bowlby (1982) stressed the importance of observing attachment “in action,” that is, behavior and thinking when the attachment system is activated. Attachment is activated when individuals or relationships are threatened, or physical or psychological safety is compromised. Strictly speaking, of course, the internal working model that is the foundation of an individual’s attachment map is not directly observable; therefore, assessment is used to activate the system to “see” the variations in its representational manifestations. The AAP reveals the client’s current responses and thoughts about attachment. Clinicians also use the AAP narratives to help uncover the details of past events.

The AAP’s depictions of major attachment events, including illness, solitude, separation, death, and threat, activate the attachment system. The AAP opens and renders amenable to interpretation those personal elements of attachment that individuals may ordinarily keep locked away and excluded from conscious awareness. Individuals make sense of the various scenes by using their perceptual and affective responses to give meaning to the picture stimuli. The external stimulus (the attachment “pull” of the pictures) initiates an internal search for applicable mental concepts, including trauma.

The AAP picture system comprises eight black-and-white line drawings. The drawings contain only enough detail to identify an event; strong facial expressions and other details are absent. The character depictions are diverse regarding culture, gender, and age. The scenes, in the order of administration, are: *Neutral* – two children play with a ball; *Child at Window* – a child looks out a window; *Departure* – an adult man and woman with suitcases stand facing each other; *Bench* – a youth sits alone on a bench; *Bed* – a child and woman sit opposite each other on the child’s bed; *Ambulance* – a woman and a child watch ambulance workers load a stretcher into an ambulance; *Cemetery* – a man stands at a headstone; *Child in Corner* – a child stands askance in a corner.

Two critical features of attachment experience are addressed in the stimulus set. One is the availability of an attachment figure. Only prompt and effective attachment figure responsiveness can successfully “terminate” attachment distress (Ainsworth et al., 1978) and create a sense of “felt security” (Sroufe and Fleeson, 1986). Infants and young children require physical proximity and access to attachment figures. Proximity and access are increasingly balanced by psychological proximity in older children, adolescents, and adults. Individuals in these older age groups can appeal to *internalized* attachment figures when attachment needs are activated, and attachment figures are absent. Some scenes portray an adult or a child alone (“alone” pictures), potentially eliciting representations of internalized attachment figures. Other AAP scenes portray adult-adult or adult-child dyads (“dyadic” pictures) that depict the physical proximity and availability of a potential attachment figure. The second critical feature is that the stimuli incorporate a lifespan perspective (Bowlby, 1982; Ainsworth, 1989). The scenes show characters that represent a range of ages, from the young child to the elderly.

The AAP is administered individually in a private setting, a therapist’s office, or a quiet space for virtual administration. The client is asked to describe what is happening in a scene, what led up to that scene, what the characters are thinking or feeling, and what happens in the end. Responses are audio-recorded and transcribed. Administration is typically 25 min. Although the pictures are potent stimuli, most individuals respond to the assessment with a cooperative attitude. They typically do not get upset during the “AAP experience” (unlike the AAI), although some may tear up or cry. On rare occasions, the interviewee asks to stop. There are clear administration guidelines to help interviewers identify defensive resistances compared with cues that would require terminating the administration session. These guidelines meet the standards for professional clinical practice.

### Coding and classification

Attachment classification using the AAP picture system analyzes the verbatim transcript of the “story” responses to the seven attachment pictures. Classification is based on coding categories we developed to evaluate the patterns and integration of three response dimensions: (1) narrative, (2) story content, and (3) defense. The following provides an overview of these dimensions. The coder evaluates these dimensions separately for each story. Of course, these features are inextricably intertwined; however, identifying the specific qualities of these features is essential to discriminating among attachment groups. There is not enough room here to provide examples and detailed descriptions. These are provided in the AAP books (George and West, 2012; George et al., 2023).

#### Narrative

The first task is to evaluate the narrative to identify portions of the response that might include personal descriptions of experience. The AAP instructions direct the individual’s attention to the depicted characters. The AAP is not a biographical interview; it does not ask the client to specify how stories are related to real life. The inclusion of personal experience indicates difficulties maintaining self-other boundaries. This tendency indicates intense distress and is often seen in the responses of individuals in the Preoccupied or Unresolved classification groups (Buchheim and George, 2011; George and West, 2012).

#### Story content

The content dimensions evaluate how the narrative conveys meaning to the relationships depicted in the storyline. *Agency of self* and *connectedness* evaluate mutuality and integrated attachment in the alone stories. *Synchrony* evaluates the combination of these features in dyadic stories.

Agency of self evidences if the capacity of the person portrayed in the picture (the projected self) takes productive steps to face the challenges introduced in the storyline (i.e., what led up to the scene). According to theory, agency best develops with consistent experiences of sensitive and responsive parental care during infancy and early childhood (Sroufe et al., 2005). In the AAP, agency of self is required to solve the problem or change the situation when facing distress or threat alone.

There are two forms of agency. The most integrated (i.e., balanced, restorative) form is when attachment figures are portrayed as a “haven of safety.” Haven of safety is depicted in themes of caregiver sensitivity to the character’s attachment need (Ainsworth et al., 1978). The other is the “internalized secure base.” Representation allows the individual to explore the inner world of the self. It is coded when the story portrays the character as drawing upon internal resources to think about distress and relationship problems. George and West (2012) developed this concept to capture the internal processes of thoughtful exploration of self, attachment figures, and events. The concept of the internalized secure base is a fundamental feature contributing to attachment security and can only be assessed using the AAP.

The other form of agency is the “capacity to act.” Capacity to act depicts the character’s ability to respond to attachment challenges or distress with constructive action. The capacity to act does not rebalance or fully assuage attachment distress, but it addresses the problem or regulates or contains the emotional response.

The alone stories are also coded for connectedness. This dimension evaluates the representational availability of intimate relationships to the alone self. Human biology defines several fundamental behavioral systems that can provide protection, including the parent (caregiving system), friends (affiliative-sociable system), and sexual-romantic relationships (sexual system) (Bowlby, 1982; Hinde, 1982; Marvin et al., 2016). The most integrated form of connectedness depicts the character reaching out to one of these fundamental relationships. By contrast, some stories depict successful connections to other people who are not part of the biological core but can be helpful (e.g., strangers or society helpers, such as doctors or police). Other stories develop themes where connections are blocked (characters fight or die). In yet other stories, the character remains alone.

Synchrony evaluates relationship quality in the dyadic pictures. Integrated synchrony is coded when interpersonal interactions depict mutual enjoyment or caregiving sensitivity. Other types of interactions portray functional behavior (e.g., giving medicine for an illness without comfort) or failures to respond (e.g., ignore the child’s bid for a hug). These responses do not qualify as integrated.

#### Defensive processes

The AAP is the only attachment assessment that shows how clients use defenses to modulate distress. Defensive processes select, exclude, and transform behavior, thought, and emotional appraisals to terminate distress as much as possible to prevent extreme discomfort or dysregulation. Attachment theory has three forms of defense: deactivation, disconnection, and segregated systems (Bowlby, 1980). Defenses are evaluated from the narrative words, images, and patterns for all seven AAP stories. Here we describe the general characteristics of each defense.

*Deactivation* shifts attention away from events or feelings that activate the attachment system and prevent the individual from becoming distracted by attendant attachment distress (Bowlby, 1980). The goal is to downplay emotions, especially anger. Deactivated contact with attachment figures serves a functional purpose; emotions are not part of the conversation. Evidence of deactivation may include themes of social rules (i.e., socially correct behavior), materialism, authority, or achievement. Interestingly, deactivation can fail to achieve the goal of neutrality and narratives “leak” underlying depictions of the self or others as negative or unworthy, expressed as themes of transgression, punishment, and rejection. Deactivating attachment defenses in the absence of integration and rebalance typify the Dismissing attachment pattern.

*Disconnection* splits attachment information and affect from the source (Bowlby, 1980). This process undermines the client’s ability to “see” and describe a unitary, consistent attachment state of mind when the attachment system is activated. Disconnection is evidenced in the story by vague, confusing, or oscillating events or feelings (e.g., good-bad, happy-depressed) story elements. Disconnection interferes with telling a unitary storyline, producing confusion and ambivalence about events and emotions. Characters are caught in cycles of waiting, wondering, and wishing for something to happen. Disconnection feeds emotions. Compared with deactivation, clients are preoccupied with emotional responses (e.g., anger, anxiety, frustration) often to the extent that they need to withdraw. Disconnecting attachment defenses in the absence of integration and rebalance typify the Preoccupied attachment pattern.

Deactivation and disconnection are “normative” regulating defenses. That is, both forms help clients regulate attachment distress. The third form of defense evidences dysregulation and attachment trauma. Bowlby (1980) called this form *segregated systems,* a concept he developed to modernize psychoanalytic repression. This extreme form of defensive exclusion develops when there is a developmental history of chronic or severe attachment threats combined with parental failed protection. Experiences and affect associated with the attachment figure and trauma (i.e., threats to broken attachment relationship or self) are “packaged up” and locked away (literally segregated) from consciousness. By activating attachment, the AAP can unleash evidence of segregated systems and trauma representations that risk emotional dysregulation. Themes may emerge in hypothetical or personal experience narratives. They include fear, helplessness, threats by others (including attachment figures), and abandonment. Segregated systems are also noted when story themes describe dangerous action (e.g., jumping out of a three-story window), or feeling out of control, or isolated. Some segregated themes include images that based on theory link unresolved attachment and dissociation (e.g., Liotti, 2017) as opposed to what is written there now.

We have found that the AAP is more sensitive to uncovering trauma than other assessments, including trauma that clients hold in a protected mental space and are reticent to discuss with others. Revealed trauma is often a source of shame. We reason that this effect is because the AAP task is not defined as a biographical report. When speaking with clients about the AAP in follow-up conversations, they often express surprise at how much we have learned about the trauma in their mental map.

## Attachment patterns: the attachment underpinnings of clients in family intervention

In this section, we describe the three traditional regulated attachment patterns –Secure, Dismissing, and Preoccupied – and three incomplete trauma patterns – Failure to Mourn (a form of Dismissing attachment), Preoccupied with Personal Suffering (a form of Preoccupied attachment), and Unresolved. Our discussion draws from several decades of attachment research that describe the nuances of each of these patterns, including expected behavior and evaluations of self and others by mothers and fathers in the context of the family system (George and Solomon, 2008, 2016; George and West, 2012; Cassidy and Shaver, 2016; George et al., 2023).

Knowing the nuances of pattern groups can facilitate clients and therapists exploring the “why” behind parents’ actions and reactions, their attributions about the motivations and emotional life of self and others, and intervention goal setting. Although a discussion of children’s contributions to family processes is beyond our current scope, we note that the AAP has been validated for adolescents as young as 13. Many clinicians use the AAP in family contexts to understand teens’ representations of attachment, reflections on parents, and other issues around autonomy seeking and relatedness that are important during this developmental period (Allen, 2008).

### Secure – flexibly integrated

The field of attachment extrapolated the term *secure* used to describe infants and children to apply to adults. Our preference is to describe this pattern as *flexibly integrated*. The reason is that there are two paths to security in adolescence and adulthood. One is a continuous path from childhood built on a foundation of sensitivity, mutual trust, flexibility, and support for emotional communication and autonomy (Ainsworth et al., 1978; George and Solomon, 2016). The other path, termed “earned secure” (Hesse, 2016), is bumpy; children do not experience the features of security listed above. Earned secure individuals work hard – often in therapy – to explore their past and why their parents acted the way they did.

Regardless of the path, the hallmark of the secure-flexible map is a rich examination of the past that creates a representation of self as worthy of attachment-figure comfort and protection and trust that parents and other attachment figures will respond in kind (Summarized in Table 1). This representational pattern on the AAP demonstrates the value of attachment-caregiving relationships as sources of reciprocal sensitivity, comfort, and mutual enjoyment. The stories show that the speaker holds images of attachment figures as present and effective in their minds when attachment is activated. The stories demonstrate the capacity to maintain boundaries – self and other, past and present; what is hypothetical and personal are distinguished. Themes of integrated agency show representations of attachment figures as accessible, comforting, sensitive to distress, and children and parents invested in relationship repair. The integrated agency in the stories often shows how rebalancing leads to constructive action (capacity to act). Secure-flexible individuals seek connections with other people, especially attachment figures and peers, when they are distressed or seek companionship. Their dyadic stories portray attachment-caregiving integration with sensitive caregiving, attunement, and mutual enjoyment. Defenses are used to support integration, relationship intimacy, and sensitivity. The AAP can show evidence of earned security, especially in those individuals whose stories include trauma indicators. Segregated systems are activated but regulated through integration that restores psychological homeostasis and demonstrates the development of confidence in relationships as the foundation of a resilient self.

**Table 1:** Secure-flexibly integrated adult attachment and parenting and family dynamics.

Secure-flexibly AAP representation	Parenting and family dynamics
Secure-flexibly integrated Attachment figures sensitive Agency: integrated; functional agency Connectedness: attachment figures and friends Synchrony: integrated Defenses support integration Trauma: resilience, repair, rebalance	Attachment: haven of safety Exploration: secure base autonomy Problem solving: balanced communications and solutions Conflict: repair, reconciliation Emotions: emotional clarity; empathy Trauma: unlikely to be triggered; seeks adult attachment figures; buffer family members from trauma

In the family system, flexibly integrated parents are committed to and enjoy being with their children. They preserve adult-child boundaries and set limits intended to guide children’s development. Established rules are subject to open discussion and negotiation. Parents serve as a haven of safety by being sensitive and valuing children’s attachment needs. Secure-flexibly integrated parents provide a secure base for exploration. The purpose of exploration is not just to learn; exploration also serves as the basis for building relationships and mutual enjoyment. The secure base fosters confidence in exploring on one’s own, knowing it is possible, and encourages a return to attachment figures for comfort and safety if exploration begins to feel uncomfortable or risky. This is especially true for fathers for whom research has shown father-child relationships emphasize exploration over comfort.

The secure-flexibly integrated parent values personal and family problems as topics for conversation. The goals of communications and actions are to find practical solutions without undermining flexibility, keeping in mind age-appropriate or situation-appropriate attachment needs and socialization demands. By maintaining adult-child boundaries, parents also create an environment that buffers children from involvement in parents’ personal or couple problems and conflicts. Problems involve potential conflict. Conflict resolution goals and outcomes for these parents demonstrate repair (i.e., rebalance, homeostasis) and reconciliation (i.e., managing discrepancies interfering with a goal). These parents value emotions and emotional communication and encourage developing a broad emotional palate. Children learn to express emotions without worrying about retribution or being squelched, including negative affect (e.g., sadness, shame, fear, anger). This quality of emotional communication creates the developmental foundation of empathy. Parents can be triggered by personal events or vicarious trauma when their children are endangered. When this happens, parents work to restore balance without turning to their children. They reach out to their adult attachment figures as havens of safety, such as their partners, parents, or professionals (e.g., therapists).

### Dismissing

The defining quality of the Dismissing pattern is defensive deactivation. Deactivation is evident as the primary form of coping throughout the AAP (Summarized in Table 2). Dismissing individuals tell stories in which they maintain firm representational boundaries – both distinctions between past and present or personal and hypothetical. Boundaries are critical components of depicting attachment figures and other adults as authority figures who give permissions, make or teach the rules of appropriate behavior, and punish transgressions. Integrated themes of agency and synchrony are rarely depicted. Rather, narratives emphasize the alone self and dyadic interaction as functional. Although distress is managed, what is missing is these narratives is the safe haven or sensitivity that only parents can provide to completely assuage attachment distress. Connectedness to others – whether adults, peers, persons in the community, or strangers – is also functional. These features of the story themes result from deactivating defenses that create distance from, minimize, reject, or avoid negative affect and outcomes. As a result, problem solving is rational (without emotions), situational, social rules that dictate behavior are paramount, and interpersonal attachment themes are deflected to emphasize achievement and personal strength. Trauma is regulated with functional agency, connectedness, synchrony, or reconciliation; representations of homeostasis and rebalancing are rare.

**Table 2:** Dismissing and failed mourning adult attachment and parenting and family dynamics.

Dismissing AAP representation	Parenting and family dynamics
Deactivated Attachment figures rejecting, distanced, functional. Firm boundaries Functional or fractured agency, connectedness, synchrony Themes: reject or disable attachment needs Trauma: regulated through functional agency, connectedness, or synchrony. No repair	Attachment: dismiss attachment needs. Functional. Authoritarian parenting. Deflect attachment needs ➔ achievement, peers, social role adults Exploration: functional, pseudo- togetherness Problem solving: rational Conflict: reject, avoid conflict Reconciliation without repair Emotions: neutralize, reject. Sympathy without empathy Trauma: repressed or dismissed
Failure to Mourn Same as Dismissing + prevalent references to trauma. Role reversal and dissociation/depersonalization risk. If regulation breaks down ➔ Unresolved pattern	Deactivation dynamics collapse ➔ Unresolved parenting, role reversal risk

Parents’ needs and rights are more significant than their children’s needs for Dismissing parents. They deactivate attachment needs by minimizing, rejecting, and ignoring them. Parents and other adults need to be inflexible authorities, set strict limits, and enforce boundaries. Bids for attachment, including emotional needs, are discouraged, and viewed as signs of weakness. Discouraging attachment is also accomplished by shifting away from relationship closeness to stress independence, achievement, and success for social status and material gain. The independence paradox in these relationships is that these parents and their children express the most separation anxiety of any other group. Parent–child activities have a quality of *pseudo-togetherness* where interactions lack emotional sharing, intimacy, and enjoyment. Problem-solving efforts are limited to the facts needed to achieve a rational solution. Emotions are unwelcome and discouraged, especially anger and sadness. There is a strong emphasis on avoiding conflict and rejecting people and situations at the source. This posture helps parents maintain an authoritarian position. When there is conflict, goals stress productive outcomes and reconciliation to avoid future disruption without repairing relationships. Because emotions are uncomfortable, dismissing parents do not encourage emotional communication, especially anger. When negative affect emerges (which is inevitable in relationships and families), it is neutralized (e.g., everybody feels that way when this happens) or rejected (e.g., you do not feel sad, you are just hungry; we do not talk about these things). Dismissing parents quickly notice misbehavior and transgressions, the source of fault, and punish. This approach to parenting undermines the development of empathy. Dismissing parents can sympathize, but only when others are deemed worthy.

In addition, Dismissing parents who experienced significant attachment trauma typically do not complete mourning and are at risk for *Failed Mourning* (*Failure to Mourn*). Mourning is blocked. This representational map shows how deactivating defenses are armor to wall off the painful threat of mourning. As Bowlby predicted (1980), Failed Mourning parents tell stories that portray images of parent–child role reversal (parentification, role inversion) because their experience is that their distraught parents are helpless to protect them. These AAPs also evidence the risk of derealization and depersonalization.

For many of the parents with trauma, deactivating strategies are so effective that they are puzzled about why the feel frustrated or sad. Our work has shown that about 50% of Failed Mourning parents cannot consistently manage their emotional state, and the Dismissing caregiving strategies we described above break down. When this happens, they become dysregulated and act like parents with Unresolved adult attachment (see below). Our work shows that it is not unusual when this happens for their children to be role reversed (i.e., the child acts like a parent) because of their sensitivity to their parents’ vulnerability until deactivating defenses restore walled off trauma.

### Preoccupied

The defining quality of the Preoccupied pattern is disconnecting defenses (Summarized in Table 3). Disconnection creates a mental fog or smoke screen that clouds the individual’s ability to create a unitary picture of attachment experience and affect. It also makes it difficult to maintain boundaries. Preoccupied individuals blur self-other distinctions, and their AAPs are more likely than the AAPs of others to digress into stories of personal experience. When stories involve attachment figures, the descriptions are often confusing because there are so many ideas about possible story themes, characters’ behaviors, and emotions. In other stories, attachment figures are portrayed as unpredictable, inaccessible (but not rejecting), or unable to decide how to respond to a child. Still other stories are laden with sentimental overtures to fill in for missing caregiving sensitivity. The smorgasbord of possibilities with no definitive outcome and confusion of the Preoccupied mental map splinters attempts to describe agency, connectedness, and synchrony. At best, these themes are functional. Often though, there is no agency or connection in alone stories. Dyadic stories often miss the point (e.g., a scared child is offered tea and cookies instead of comfort). Stories are more likely to be emotional than the stories of Dismissing individuals. Emotions are heightened and entangling. Commonly, individuals attempt to disconnect from trauma with euphemisms (e.g., something “horrible” happened, the child is trying to manage something “hard and heavy”).

**Table 3:** Preoccupied and preoccupied with personal suffering adult attachment and parenting and family dynamics.

Preoccupied AAP representation	Parenting and family dynamics
Preoccupied – disconnected Attachment figures unpredictable, confused, palliative. Blurred boundaries Agency, connectedness, and synchrony are functional, fractured, or absent. Themes: confused, emotionally preoccupied and entangled Trauma: fractured regulation through functional agency, connectedness, or synchrony	Attachment: confused, inconsistent, noncontingent responsiveness. Permissive parenting. Blurred boundaries. Disconnect from/tune out distress Exploration: enjoys, encourages immaturity and dependency Problem solving: fairness, guilt driven Conflict: deflect, circumvent, exaggerate emotions Emotions: heightened ➔ moody, worry, anger, frustration, guilt. Sentimental without empathy. Trauma: confused
Preoccupied with personal suffering Same as preoccupied + frequent references to trauma. Dissociation or depersonalization risk. Fragile regulation	Same as Preoccupied.

Children’s rights are more significant than the parents’ needs for Preoccupied parents. Parents value emotional intimacy and happiness, seemingly at all costs. Attention to children’s attachment cues is heightened, contributing to an undercurrent of anxiety and tension. Given the confusion in the AAP, it should not be surprising that these parents are confused about how to read situations and what to do about them, which for some end up as complaints of being stressed and exhausted. Yet when given a chance to “get away” from children, they lament that they cannot wait to get home. Their behavior can be unpredictable, noncontingent, guilt-ridden, embarrassing, or absent all-together. These parents encourage and enjoy dependency. Confused about the best strategies to protect children, they want to keep them physically and emotionally close “to the nest” at the cost of age-appropriate exploration. Problem-solving focuses on fairness and family equality. Emotions are welcome but poorly differentiated. Empathy for others is confounded by blurred boundaries that have trouble differentiating the needs of individual family members. Preoccupied parents try to circumvent conflict by changing the subject or distraction. Emotions are heightened since they are so important in these relationships. Without clear emotional communication, however, parents and children are flustered and complain about emotional ambiguity. Parents, children, and other family members are often seen as inexplicably moody, worried, or frustrated. Anger, guilt, and shame are poorly managed because of lingering unaddressed emotional residues.

Preoccupied individuals with childhood trauma are at risk for chronic incomplete mourning, called *Preoccupation with Personal Suffering.* We see in their AAP representational map attempts to remove or blur the effects of trauma with vague aspirations that someone will come along and help or magical thinking that somehow the character will survive without being able to describe how this happens. These AAPs also show evidence of derealization and depersonalization.

Our work has shown that preoccupied parents and preoccupied sufferers engage similarly with their children. The smoke screens surrounding trauma created by disconnecting defenses appear to effectively diffuse the potential for caregiving breakdown and keep these parents involved. Hope and magical thinking also help out when they are triggered.

### Unresolved

Unresolved attachment can have elements similar to any other patterns (Summarized in Table 4). The defining quality of the Unresolved map is the inability to regulate trauma. This failure creates a state of mind where terrifying memories and emotions threaten to flood consciousness. The Unresolved pattern shows the greatest tendency for personal experience intrusions in the AAP narratives, including their trauma. Deactivating and disconnecting defenses, and agency, connectedness, and synchrony break down, and themes of trauma that were segregated invade the narrative. There are two Unresolved AAP responses. One is a flooded and dysregulated narrative (i.e., trauma is not managed or contained); the other is a constricted response where the individual freezes up and cannot describe anything to the picture stimulus. Constriction is a mammalian fear response that freezes thoughts, feelings, and actions so they cannot be “seen.” Constriction is the antidote for flooding.

**Table 4:** Unresolved adult attachment and parenting and family dynamics.

Unresolved AAP representation	Parenting and family dynamics
Unresolved - dysregulated segregated trauma AAP patterns could be like Secure-Flexible, Dismissing, or Preoccupied. Risk of intrusion of personal traumatic experiences Segregated system defenses and trauma themes: fear, isolation, helpless, loss, abuse Flooded – dysregulated, not contained Constricted – cannot respond	Flooded Attachment: abdicate, fail to protect Exploration: risky, unmonitored Problem solving: controlling strategies Conflict: combative Emotions: frightened, emotionally intelligent Trauma: overwhelmed. Risk of re-enacting trauma Constricted Attachment: role-reversed Exploration: frozen, cannot explore Problem solving: role reversed Emotions: emotional merging Trauma: dissociation or depersonalization risk

In low-stress family interactions, Unresolved parents approach parenting in ways similar to any of the three regulated patterns we described above. However, these parents are chronically at risk of being triggered and re-enacting their trauma and experiences. The stress triggers can be low and idiosyncratic. Unresolved parents become frightened, overwhelmed, and helpless. Their children experience their attachment figures as turning away and abdicating the fundamental protective function of caregiving. This failure is most visible for flooded parents. Failure risks dangerous situations within or outside the family without parental remedy. Children come to understand at an early age that they must manage on their own. Without a haven of safety or a secure base, the children of Unresolved parents are anxious, hypervigilant, and risk engaging in reckless activities without regard to threat. Internally frightened because they cannot protect themselves, these children develop external strategies to dominate and control their parents and environment. These strategies may appear to others as independence and leadership; however, the psychological undercurrent motivating these strategies are brittle attempts at self-protection and controlling the people around them and their environments. Unresolved parents develop controlling behavior for these same reasons – internal helplessness and fear of failed protection. When both interactive partners are desperate for control, conflict and arguments in these dyads become combative battles that aim to win, not resolve problems. In summary, flooded by unmetabolized trauma, this pattern of Unresolved attachment injects emotional dysregulation and chaos into family life.

The pattern for constricted Unresolved parents is qualitatively different. Similarly helpless, these parents shut down. They appear vulnerable, childlike, immature, and are at risk of physically or psychologically disappearing (e.g., dissociation risk). Their children, then, take on the caregiving safe haven responsibility to fortify parents and nurture them back to the role of the stronger and more caring person in the relationship. Like their parents, these children are frightened, but the role reversal directed toward their parents is mutually nurturing and helps rebuild the relationship. As with children of flooded parents, the children of constricted parents do not explore. These parents do not create the secure base-safe haven dynamic that supports exploration. The children of constricted parents can appear frozen, not curious, and unwilling to risk launching away from their parents or the home because they are frightened and hypervigilant to danger. Our work shows that constricted parents do not report many family problems, likely because role reversed children remedy potential conflict. Parents tell us that their children are so precocious, sweet, empathic, and attuned to the emotional life and circumstances of others that problems rarely occur. In addition, many children take on the roles of comedian or clown, a phenomenon we have observed as early as toddlerhood. Keeping the parent happy through laughter is a well-received way to control a relationship (as opposed to combative punitive behavior). These parents and their children are very empathic toward others. Our work shows that the source of their empathy is boundary dissolution where parents and children are emotionally merged. Sadness and concern for self, equate to sadness and concern for the other family and community members.

## Using the AAP in family therapy

The versatility of the AAP lends itself to a wide range of ways it can be used in therapy to help move parents, children, and the family system in the direction of a secure base for exploration of individual and relationship dynamics. Our purpose in this section is to provide the reader with a few examples of how the therapist may use the AAP with the family members who tell these stories to understand themselves and relational functioning.

Earlier we presented details about each attachment and incomplete pathological mourning group. Knowing the AAP attachment group can help the therapist pinpoint and anticipate where many of the family problems originate. In some situations, questions may center on why parents are not working well as a dyad and as coparents; attachment assessment reveals the layers that explain why. Consider the example of a family with one Dismissing parent and one Preoccupied parent (a common co-parent variation). Both parents are vulnerable and insecure; they did not get their attachment needs met in childhood and are poorly poised to provide for their children’s attachment needs. However, their attachment maps for interacting with each other and their children are diametrically opposed. We can expect to see these differences expressed in how they view themselves and family relationships. The Dismissing parent was raised to reject vulnerability and stress relationship distance, independence, and rationality. We would expect to observe this parent to reject their partner’s and their children’s attachment needs, pushing independence, rational problem solving, and especially for children, achievement success. The Preoccupied parent was raised in an environment of entangled relationships, emotionality, confusion, and dependency. We would expect to observe this parent to seek intimacy and closeness beyond their partner’s comfort zone and have trouble deciding on parenting goals and strategies. We would not be surprised that this parent radiates guilt, worry, and frustration, which is unacceptable to their Dismissing partner. Children develop different relationships with different parents. The children in this family would be caught in a system contradiction, having to straddle different expectations from each parent and not getting their attachment needs fully met by either of them. In clinical work, it is not unusual for us to work with families where one or both parents have experienced childhood trauma. The AAP provides insight as to how and to what degree parents have completed mourning. The common challenge for these adults as parents and partners is their childhood experiences of parental failed protection. The nuances in their AAP are key to observing and understanding how different defensive maps regarding failed protection plays out in family dynamics. Thus, by highlighting the family at the dyadic levels, we can observe how knowing the attachment representations in addition to the AAP content and defenses can help us identify how partner, coparent, and parent–child dances are not functioning. The AAP both provides a snapshot of how parents’ attachment representations lie at the core of family difficulties and cascade to these other relationships, and provides insight for how family therapy can address each of these relationships to align in the direction of secure base functioning.

The AAP stories are powerful tools in and of themselves. We look “inside” the stories to get a clearer picture of the interaction of content and defensive processes than depending on the classification group alone. For example, LeBlond et al. (2023) described using the AAP to better inform the care provided to adolescents with a kidney transplant from a parent donor. The adolescent patient’s AAP in their case example was Unresolved. The AAP stories identified the specific nature of this patient’s dysregulating problems, which were feeling isolated, parental failed protection, and fears of abandonment and death. These authors stressed how important uncovering the specifics of this patient’s fears was for treatment as well as recognizing the effect of the father’s denial of his child’s risks, which included death. The patient’s AAP stories negated their interview narrative of joy and gratitude toward the father by exposing his unconscious fears. The AAP uncovered the “psychological and impact of living with chronic disease,” (p. 176) that was unspoken and denied. Similarly, Mazzeschi et al. (2023) described the power of incorporating the AAP in family Therapeutic Assessment for treatment of childhood obesity. The authors explained how the AAP assessment had identified the parents and the patient all revealing some form of incomplete pathological mourning. The parents were Unresolved, and the adolescent patient was Failed Mourning. They explained how knowing the parent’s AAP map informed the therapist about the care needed to buffer the parents from becoming overwhelmed by their attachment fears while discussing their helplessness and fears surrounding helping their child. The therapist also saw from the patient’s AAP that they had created deactivated armor to block becoming flooded by fear and helplessness. The AAP also showed that the patient viewed their parents as rejecting and unable to see or respond to their distress about their condition. The therapist’s goal was to “interrupt the cycle of unconscious activation of [their] fears and worries and begin to address them directly” (p. 198) to move the patient in the direction of mourning and family change.

Clients often are amenable to change when they are helped to see and name their attachment-related strengths and wounds in the AAP stories. Therapists can select single stories, for example, to explore with their clients. We describe in this example a traumatized adolescent client whose stories evidenced intense attachment trauma throughout the AAP. What stood out to the therapist was one particular trauma story that portrayed parentified role reversal, a nuance that had not been expressed in earlier therapist-client discussions. The therapist read this story aloud with the client, including the attachment-theory interpretation of what the story meant. The client broke down, sobbing and affirming a secret that had never before been spoken; they described how overwhelming it had been to be in the position to take care of others their whole life. The therapist later read this story to the client’s parents, who never before realized their child’s parentification or knew the burden their child carried of failed protection and role reversed caregiving.

## Conclusion

Across many, if not most therapeutic approaches, the non-specific factors that the therapist brings are thought to account for a percentage of change (Priebe et al., 2020). The attachment approach of conceptualizing the therapist as a secure base from which to explore difficulties in therapy builds on this idea. The therapist in this manner is a key element of change through the role they are able to play in a providing secure relationship for the families they treat. In turn, the therapist supports parents to become the secure base and safe haven in their families.

Although some family therapists have used attachment theory as a basis for thinking about family difficulties, the ability to implement the nuances of this approach requires assessing attachment representations among family members to better understand *specific* family dynamics. Assessment has been a significant barrier. The AAP provides an efficient yet rich approach to assessing parents’ attachment representations that therapists can use to guide their understanding of the complex interplay of family relationships. Extensive research regarding the attachment and caregiving systems provides predictions regarding individuals’ behaviors that allows therapists to quickly pinpoint likely sources of family difficulties and potential targets for intervention. The AAP may provide therapists with a perspective on family dynamics that might otherwise take substantial information gathering to inform the therapeutic framework and approach.

The AAP is an easy economical measure to use clinically. Therapists would administer it individually to adult and adolescent family members. Trainings are available to teach the coding and classification system. Following training, therapists are encouraged to engage in a reliability process to ensure the quality of AAP interpretation and ethical use. There are also resources for trained therapists to have their clients’ AAPs coded by master judges. Many therapists join consultation groups comprised of AAP users to learn about others’ views, interpretations, and therapeutic approaches when using the AAP with their clients. For more information, the interested reader is referred to the AAP website – www.attachmentprojective.com.

## Data availability statement

The original contributions presented in the study are included in the article/supplementary material, further inquiries can be directed to the corresponding author.

## Author contributions

CG: Conceptualization, Writing – original draft. JW: Conceptualization, Funding acquisition, Writing – review & editing.
